# Problem Drinking, Alcohol-Related Violence, and Homelessness among Youth Living in the Slums of Kampala, Uganda

**DOI:** 10.3390/ijerph15061061

**Published:** 2018-05-24

**Authors:** Monica H. Swahn, Rachel Culbreth, Nazarius Mbona Tumwesigye, Volkan Topalli, Eric Wright, Rogers Kasirye

**Affiliations:** 1 Division of Epidemiology and Biostatistics, School of Public Health, Georgia State University, P.O. Box 3984 Atlanta, GA 30302-3984, USA; rculbreth@student.gsu.edu; 2 Department of Epidemiology and Biostatistics, School of Public Health, Makerere University, Kampala 00256, Uganda; naz@musph.ac.ug; 3 Department of Criminal Justice and Criminology, Andrew Young School of Policy Studies, Georgia State University, P.O. Box 3992, Atlanta, GA 30302-3992, USA; vtopalli@gsu.edu; 4 Department of Sociology, College of Arts and Science, Georgia State University, P.O. Box 5020, Atlanta, GA 30302-5020, USA; ewright28@gsu.edu; 5 Uganda Youth Developmental Link, P.O. Box 12659, Kampala 00256, Uganda; kasiryer@yahoo.com

**Keywords:** homelessness, alcohol use, youth violence

## Abstract

This paper examines problem drinking, alcohol-related violence, and homelessness among youth living in the slums of Kampala—an understudied population at high-risk for both alcohol use and violence. This study is based on a cross-sectional survey conducted in 2014 with youth living in the slums and streets of Kampala, Uganda (*n* = 1134), who were attending Uganda Youth Development Link drop-in centers. The analyses for this paper were restricted to youth who reported current alcohol consumption (*n* = 346). Problem drinking patterns were assessed among youth involved in alcohol-related violence. Mediation analyses were conducted to examine the impact of homelessness on alcohol-related violence through different measures of problem drinking. Nearly 46% of youth who consumed alcohol were involved in alcohol-related violence. Problem drinkers were more likely to report getting in an accident (χ^2^ = 6.8, *df* = 1, *p* = 0.009), having serious problems with parents (χ^2^ = 21.1, *df* = 1, *p* < 0.0001) and friends (χ^2^ = 18.2, *df* = 1, *p* < 0.0001), being a victim of robbery (χ^2^ = 8.8, *df* = 1, *p* = 0.003), and going to a hospital (χ^2^ = 15.6, *df* = 1, *p* < 0.0001). For the mediation analyses, statistically significant models were observed for frequent drinking, heavy drinking, and drunkenness. Interventions should focus on delaying and reducing alcohol use in this high-risk population.

## 1. Introduction

Alcohol use is a widely known risk factor for involvement in many types of violence, but specifically for youth violence, both as a perpetrator and victim [1,2,3,4]. Unlike other forms of violence that usually have a very specific definition based on the victim-perpetrator relationship, youth violence can include assaults which may occur in bars, nightclubs, or on the streets, but may also include bullying, sexual aggression, and gang-related violence with less consideration for the victim-perpetrator relationship [1]. While youth violence and its morbidity are difficult to measure across cultures and countries, the global homicide rate of 6.7 per 100,000 [5] underscore the significant public health burden represented by violence. Specifically, alcohol is estimated to be responsible for 26% and 16% of life-years lost due to homicide among males and females, respectively [1]. Moreover, several population-based studies have shown a correlation between population-level alcohol consumption and violence rates [6,7] and also between stricter alcohol policies and lower homicide levels [8,9,10]. However, there is substantial variability across settings with less attention and research on the prevalence of problem drinking, alcohol-related violence, and its risk factors in developing countries, particularly in sub-Saharan Africa. Nationally representative samples of high school students have shown high levels of problem drinking in both Zambia (45.1%) and Uganda (21.5%) [11], as well as among youth in Burkina Faso, Malawi, Ghana [12], South Africa and Tanzania [13,14]. While these studies document a high prevalence of problem drinking in specific African countries, research on youth alcohol use and alcohol-related violence among vulnerable populations remains scarce in sub-Saharan Africa, specifically in Uganda.

Research suggests that alcohol affects cognitive abilities, disinhibition, lack of self-control, and increased impulsivity, which in turn contributes to violence [1,15,16]. In fact, the association between alcohol use and violence has been classified as a dose-response relationship, where higher levels of alcohol use correspond to higher rates of violence [1,15,16,17,18]. Typically, risky drinking patterns such as high-volume drinking, binge drinking, frequent drinking, or other forms of excessive alcohol use have been found to be most strongly associated with violence and other forms of alcohol harm, such as risky sexual behaviors, unintended pregnancies, sexually transmitted infections (STIs), and delinquency [17,19,20,21]. The conceptualization of problem drinking and alcohol-related violence is informed by Bandura’s social learning theory, which states that individuals’ behavior is a function of environmental and individual traits [22,23]. Observational learning may also be a factor in parental alcohol use and youth engagement in alcohol use. Additionally, the link between problem drinking and aggression among homeless youth could also be directly related to observational learning, where individuals who are living without shelter may resort to alcohol use and problem drinking as a coping skill, thereby influencing other youth in the environment [22,23,24].

Recent research has broadened the scope of inquiry of alcohol-related violence to examine not only the individual-level factors that have been strongly linked to youth violence, such as a heavy drinking history [25], delinquency and poor school performance [17], family education level [17], and social or peer pressure [25], but also the larger social context. An intriguing new area of research has focused more specifically on the social context to capture social disadvantage in likely vulnerable populations. For example, one relatively recent study found that acute alcohol intoxication significantly increased aggressive behaviors (as measured in a laboratory experiment using a modified version of the Taylor Aggression paradigm [26]) specifically among adult participants who grew up in disadvantaged neighborhoods [27]. In this case, disadvantaged neighborhoods were defined through measures of vandalism, prostitution, and other social disorganization characteristics using the “Neighborhood Scale” developed previously [28]. The authors concluded that this experimental study supported the notion that “legacies of neighborhood inequality” [29] have implications for violence prevention in real world settings [25].

The extent to which neighborhood disadvantage may contribute to alcohol-related violence among vulnerable youth in Sub-Saharan Africa has not been examined. However, countries in this region are often typified by localized poverty and structural inequality. Growing up in a disadvantaged community or neighborhood has been associated with an increased risk of experiencing violence and aggression [25,30]. Moreover, alcohol use is also highly prevalent in disadvantaged neighborhoods [31]. Roche and colleagues for example, examined alcohol use and alcohol-related harms comparing high socioeconomic groups to low socioeconomic groups and found that for the same alcohol use levels, individuals with a lower socioeconomic status experienced more alcohol-related harms than those with a higher socioeconomic status [32]. Thus, it is likely that neighborhood context may be related to both alcohol use and to involvement in violence. 

In Kampala, Uganda, youth who live in disadvantaged neighborhoods, specifically in the slums, are at a high risk for a broad range of adverse health outcomes, including problematic alcohol use [33], violence victimization and perpetration [33,34], sexual violence victimization [35], HIV [35,36], suicide ideation [37], and homelessness [33]. While the youth who live in the slums may all experience hardship and community disadvantage, it is possible that those who report having been homeless for some period of time face the most severe forms of disadvantage [24,38,39,40]. Slums are typically classified as areas lacking government planning and infrastructure that is manifested through overcrowding, a lack of access to social services, and poor environmental, social, and economic conditions [41]. Homeless youth, defined as those sleeping strictly on the streets and not in a shelter, face compounding risks associated with social exclusion [42,43,44], access to alcohol [45], and lack of access to social justice resources [46]. Homeless youth have also been found to report higher levels of psychological distress and mental health problems, experience more significant emotional and physical traumas, struggle more to maintain supportive networks, and have higher rates of substance use and abuse than youth who live with their families or live in shelters [47,48,49,50,51]. However, scientific understanding of how these factors are related in the lives of homeless youth remains limited. As such, it is important to determine the extent to which a history of homelessness may be a risk factor for alcohol-related violence, but also to determine whether problem drinking and risky drinking patterns may mediate that association. 

Most of the research on problem drinking, alcohol-related violence, and homelessness has been conducted in the U.S. and other developed countries. No study, to our knowledge, has examined problem drinking, alcohol-related violence, and homelessness among youth living in the slums of Kampala—an understudied population at high risk for both alcohol use [33] and violence [34]. The current study seeks to fill an important research gap by examining problem drinking and risky drinking patterns, alcohol-related violence, and homelessness among youth living in the slums of Kampala. The research questions that guided this work were: (1) what are the demographic characteristics and psychosocial correlates of alcohol-related violence among youth living in the slums of Kampala?; (2) what proportion of youth involved in violence are also problem drinkers?; and (3) does problem drinking mediate the association between homelessness and alcohol-related youth violence? The answers to these questions can guide prevention and intervention efforts that seek to prevent and reduce alcohol-related harm by identifying the specific and modifiable risk factors for alcohol-related violence. 

## 2. Materials and Methods 

### 2.1. Setting

The current study is based on the “Kampala Youth Survey 2014”, a cross-sectional survey conducted in March and April 2014 to quantify high-risk behaviors and exposures, with a focus on alcohol use, sexual behaviors, and HIV, among urban youth, 12–18 years of age, living in the slums or on the streets of Kampala, Uganda, who were participating in a Uganda Youth Development Link (UYDEL) drop-in center for disadvantaged street and slum youth [52]. Study participants were recruited at six drop-in centers and the neighborhoods surrounding the UYDEL drop-in centers primarily through word of mouth.

### 2.2. Data Collection

Over the 15-day (19 March to 2 April) data collection period, 1628 youth were approached by a social worker or peer educator to participate in the survey which occurred at multiple sites. Among these youth, 131 declined yielding a participation rate of 92%. A total of 1497 surveys were collected. Due to technical issues with an offline server, 320 surveys were lost, yielding a final analytic sample of 1134 surveys (56% girls). 

Each social worker/peer educator received training on the study methodology and recruited potential participants among attendants at the drop-in centers and surrounding neighborhoods. Peer educators and social workers conducted the face-to-face interviews, and each of the questions were translated into Luganda (local language) if necessary. Participants could also select to take the entire survey in Luganda (*n* = 4). Regardless of the language selected, the survey was administered to the participants on electronic tablets, which allowed for easier administration. Participants were informed about the study and read (or were read) the consent forms, before providing verbal consent to participate in the study. Youth who “cater for their own livelihood” are considered emancipated in Uganda and are able to provide their own consent for the survey without parental consent. Participation was limited to youth ages 12–18 present in-person during the data collection period. There were no other exclusion criteria. Recruited youth received a small snack as an incentive for participating in the survey. Institutional Review Board (IRB) approvals were obtained from Georgia State University and the Uganda National Council for Science and Technology to conduct this study in Kampala (SS3338).

The Kampala Youth Survey 2014 was mostly based on previously validated quantitative measures to assess alcohol use, violence perpetration and victimization, prevalence of alcohol marketing, sexual behaviors, history of HIV/AIDS and other STIs, and mental health among adolescents. The previously validated measures were obtained from surveys conducted in the U.S. and globally, including: Global School-based Student Health Survey (GSHS) [53], Kampala Youth Survey 2011 [33,34,36], MAMPA (Monitoring Alcohol Marketing in Africa) 2012 Questionnaire, AUDIT (Alcohol Use Disorders Identification Test) Questionnaire [54], CAGE (Cut-Annoyed-Guilty-Eye) Questionnaire [55], iMPPACS, AIDS Indicator Survey [56], and the Demographic Health Survey [57].

### 2.3. Data Analysis

Demographic characteristics including gender, age, and education were measured among the youth. All analyses were restricted to youth who reported consuming alcohol in the past 12 months (*n* = 346). The original study was powered to detect a minimum number of youth who consumed alcohol, along with other high-risk behaviors, such as risky sexual behaviors and violence-related behaviors. Alcohol-related violence, the main outcome, was measured using: “Because of your own alcohol use, how often during the last 12 months have you experienced the following–Got in a fight?” Participants could answer “Never,” “1–2 times,” or “3 or more times.” For the purpose of this paper, responses were dichotomized into “No” and “Yes” to indicate any physical fighting due to alcohol use. Specifically, we were interested in examining the outcome of whether alcohol-related physical violence was reported, not the number of occurrences. Therefore, this variable was dichotomized to better answer our intended research question. Youth who responded “Yes” to alcohol-related physical violence were classified as engaging in alcohol-related violence, and youth who responded “No” to alcohol-related physical violence were classified as not engaging in alcohol-related violence. Psychosocial measures also included experiencing childhood abuse and parental alcohol use. Childhood abuse was measured using: “Did your parents ever beat you so hard you had bruises or marks?” Participants could answer “Yes” or “No.” Additionally, parental alcohol use was measured using: “Did your parents/caretakers drink a lot of alcohol when you were growing up?” Participants could answer “Yes” or “No.” Chi-Square tests were performed to examine these differences. For the only continuous variable age, the distribution was non-normal, and thus, differences between alcohol-related violence and no alcohol-related violence was determined using the Wilcoxon Rank-Sum Test. 

Other alcohol-related violence was also assessed among problem drinkers and non-problem drinkers. Other alcohol-related violence included measures which asked youth if they got in a fight, got in an accident, had serious problems with their parents, had serious problems with their friends, was a victim of robbery or theft, had trouble with the police, or had to go to a hospital due to their own alcohol use in the past 12 months. Problem drinkers were classified using CAGE scores [55]. The CAGE questionnaire consists of questions, such as: “Have you ever felt you should cut down on your drinking?”; “Have people annoyed you by criticizing your drinking?”; “Have you ever felt bad or guilty about your drinking?”; and “Have you ever had a drink first thing in the morning to steady your nerves or to get rid of a hangover (eye opener)?” [55]. Items are scored 0 or 1, and a higher score indicates a more severe alcohol problem. Scores are totaled and scores of 2 or more are considered “clinically significant,” and therefore, classified as problem drinkers [55]. The CAGE questionnaire has previously been tested with good test-retest reliability (0.80–0.95) [58]. Additionally, the CAGE questionnaire has been used in several countries in Eastern Africa to assess problem drinking [58]. Chi-Square tests were computed to determine differences in problem drinkers compared to non-problem drinkers for other alcohol-related violence measures. Metrics of risky drinking that were included in mediation models were operationalized as follows: problem drinking (CAGE scores > 2); frequent drinking (alcohol frequency 5 or more times a month); heavy drinking (3 or more drinks consumed on a typical day); binge drinking (drinking 5 or more drinks on one or more days in the past month); and drunkenness (drunk on one or more days in the past month). Additionally, homelessness was measured using: “Have you ever lived on the streets with nowhere else to go?”

Lastly, five separate mediation models were computed to determine if various risky drinking patterns mediated the association between homelessness and alcohol-related physical violence. Multiple problem drinking measures were examined. While all youth were living in the slums of Kampala, we were interested in examining a history of homelessness specifically (i.e., living on the streets and not living in a shelter or with family) compared to youth who were not homeless. Mediation models were computed accounting for categorical variables and utilized bootstrapping methods (*n* = 5000) to construct confidence intervals for indirect effects. All of the paths were simultaneously estimated in each model, and each of the five mediation models were examined separately. A significance level for all analyses was set a priori at 0.05. All analyses were conducted in SAS 9.4 (SAS Institute, Cary, NC, USA) and Mplus 7.0 (Muthén & Muthén, Los Angeles, CA, USA). 

## 3. Results

Among youth who participated in the study and reported current alcohol use (*n* = 346), nearly 46% were involved in alcohol-related violence (Table 1). Among youth who reported alcohol-related violence, 50% were female, 75% reported parental alcohol use, nearly half reported ever living on the street (49%), and 60% reported problem drinking as defined by the CAGE scores. Alcohol-related violence was statistically significantly associated with parental alcohol use (χ^2^ = 4.6, *df* = 1, *p* = 0.03), parental abuse of youth (χ^2^ = 6.9, *df* = 1, *p* = 0.009), ever living on the streets (χ^2^ = 13.9, *df* = 1, *p* = 0.0002), and problem drinking (CAGE scores) (χ^2^ = 16.9, *df* = 1, *p* < 0.0001). Other measures of risky alcohol use, including alcohol use frequency, number of drinks per day, binge drinking days in the past month, and number of drunk days in the past month were also associated with alcohol-related violence. 

Alcohol-related harm comparing problem drinkers to non-problem drinkers are presented in Table 2. Problem drinkers were more likely to report getting in an accident (χ^2^ = 6.8, *df* = 1, *p* = 0.009), having serious problems with parents (χ^2^ = 21.1, *df* = 1, *p* < 0.0001), having serious problems with friends (χ^2^ = 18.2, *df* = 1, *p* < 0.0001), being a victim of robbery or theft (χ^2^ = 8.8, *df* = 1, *p* = 0.003), and going to a hospital (χ^2^ = 15.6, *df* = 1, *p* < 0.0001). 

**Figure 1:**
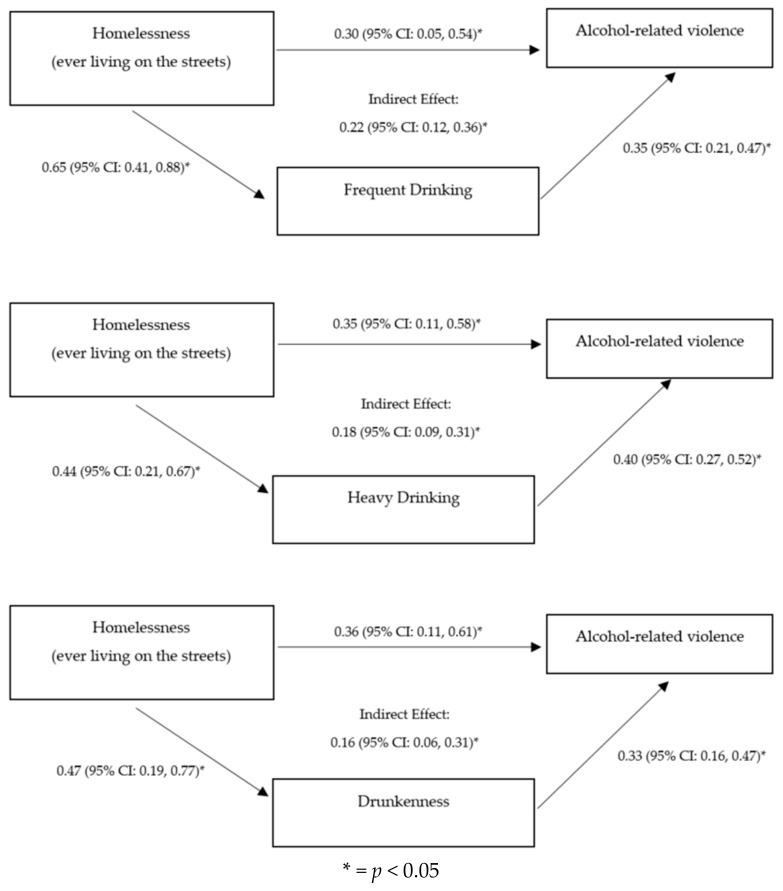
Mediation models for homelessness, problem drinking/risky drinking patterns, and alcohol-related violence among youth living in the slums of Kampala who reported alcohol consumption in the past year (*n* = 346).

Five models were conducted to examine the mediation of problem drinking on the association between homelessness and alcohol-related physical violence. Statistically significant mediation models are presented in Figure 1. All analyses were restricted only to current drinkers, as in the previous tables. Problem drinking defined by CAGE scores and binge drinking were not statistically significant mediators. However, statistically significant models were observed for frequent drinking (Indirect effect: 0.22; 95% CI: 0.12, 0.36), heavy drinking (Indirect effect: 0.18; 95% CI: 0.09, 0.31), and drunkenness (Indirect effect: 0.16; 95% CI: 0.06, 0.31).

## 4. Discussion

In this study we examined factors associated with alcohol-related violence among youth living in the slums of Kampala. The findings show that parental alcohol use, childhood abuse, homelessness, and frequent, heavy drinking and drunkenness and problem drinking measured by the CAGE were significantly associated with alcohol-related violence. The most surprising finding was that both female and male youth were equally represented among those reporting alcohol-related violence. While we do not know the context for the alcohol-related violence disclosed, it is generally believed that girls are less likely than boys to engage in youth violence [59,60]. Additionally, previous research in the U.S. has shown that there is a substantially lower prevalence of alcohol-related violence among adolescent girls (8.0%) than adolescent boys (15.6%) [17]. Other underlying risk factors may account for the surprisingly high prevalence of alcohol-related violence among girls. For example, youth living in the slums of Kampala have a high prevalence of involvement in commercial sex work (13.7% among sexually active youth), and sex work has been found to be strongly associated with both alcohol use and violence victimization specifically [36]. As such, this study underscores the need to determine the modifiable risk factors for young women who may engage or be subjected to alcohol-related violence. 

This study also sought to examine the types of alcohol-related harm reported by those categorized as problem drinkers by their CAGE scores versus those that were not problem drinkers. Since these analyses were limited to youth who reported current alcohol use, these findings indicate that youth who report problem drinking also report a higher prevalence of alcohol-related harm compared to youth who reported drinking but were not classified as “problem drinkers” based on their CAGE scores. Specifically, these comparisons show that problem drinkers were more likely than non-problem drinkers to report getting in a fight, getting in an accident, having serious problems with parents, having serious problems with friends, having been robbed, or been to the hospital for an alcohol-related injury or illness. These findings underscore the need to address and reduce the alcohol harm experienced by these youths. In Uganda, the legal drinking age is 18. However, the mean age of the drinkers in this study was 17 years, indicating that most were underage with respect to the legal drinking age. Primary prevention efforts to prevent and reduce alcohol use among homeless underage youth would be a key recommendation, with a particular focus on addressing factors unique to homelessness, such as dire environmental living conditions, food and money scarcity, and mental health concerns. Moreover, while our findings demonstrate a range of alcohol-related problems including violence among problem drinkers, the context for these problems cannot be assessed in this study. But, these findings clearly indicate the need for future research to better understand the context in which homeless youth drink and how problem drinking can be addressed and reduced to prevent further harm among these marginalized youths.

Our key finding also show that among youth living in the slums of Kampala, problem drinking was strongly associated with alcohol-related violence. This association is consistent with the Social Learning Theory [22]. Our previous research has demonstrated that alcohol use is more common among those who have been physically abused [33] and that parental alcohol use was highly associated with physical abuse as well as parental partner violence [33]. As such, it is not surprising that those youths who reported alcohol-related violence were also more likely to report parental alcohol use and prior childhood abuse. 

We operationalized problem drinking in five different ways based on previous literature [55,56,61]. In this study, problem drinking was measured by “frequent drinking”, “heavy drinking”, and “drunkenness”, and each were statistically significant mediators between homelessness and alcohol-related violence. These results are consistent with the growing literature linking problematic alcohol use and alcohol-related violence (see References [62,63,64]), and also demonstrate that problem drinking is key among those reporting homelessness and living in the most disadvantaged of circumstances. However, our findings show that problem drinking, as defined by the CAGE score, and binge drinking were not statistically significant mediators. It is possible that frequent drinking, heavy drinking, and drunkenness measures capture more of a problematic alcohol use construct compared to the CAGE scores and binge drinking measures, which would explain the lack of statistical significance found between the CAGE scores and binge drinking measures with alcohol-related violence. Frequent drinking may expose the youth to more opportunities to engage in alcohol-related violence. Similarly, drunkenness, which measures the number of days drunk in the past month, would also increase the opportunities for alcohol-related violence. Additionally, overuse of alcohol is associated with disinhibition, potentially leading to increased aggression and impulsivity related to violence [1,15,16]. 

Several limitations should be considered while interpreting the findings from this study. More specifically, due to the cross-sectional nature of this study, causal mechanisms cannot be assumed or inferred between homelessness, problem drinking, and alcohol-related violence or any other associations reported. Additionally, the timeframe of the multiple variables and outcomes examined may overlap and therefore make it impossible to establish the sequencing and timeline of these experiences. Homelessness was also assessed as having a history of being homeless, rather than current homelessness. Additionally, our measure of alcohol-related violence only asked about “alcohol-related fighting” with no other questions regarding the circumstances or the context for the aggressive behaviors. Alcohol-related fighting could be attributed to many different contexts, including the possibility of youth engaging in self-defense to protect themselves or their property. Moreover, due to the sensitive topics discussed, social desirability bias and misclassification may have also produced an underestimate of the overall and true prevalence of alcohol-related violence in this population. Finally, the convenience sample of adolescents surveyed for this study was also limiting. However, it should also be noted that this population is hard-to-reach and a clear sampling frame does not exist, thus, the successful recruitment of a large sample was a strength of this study. These limitations, while important, are mitigated by the scarcity of empirical findings in this and similar populations related to problem drinking and alcohol-related harm including violence. It is our hope that these findings may be used to generate new research to address many of the health concerns they face and to prevent youth violence. Specifically, future research should investigate the context of alcohol-related violence to further inform targeted interventions. Future studies should examine the comparisons of frequent problematic alcohol use and the severity of problematic alcohol use with alcohol-related violence in this population. Additional research should also examine potential underlying factors for the high prevalence of alcohol-related violence and fighting among girls.

Research on youth living in slums across sub-Saharan Africa is limited. However, the continued migration to slums, which are expanding rapidly [65], underscore that public health research is needed to understand and mitigate the risk that these youth face, not only for violence, but also for other adverse health outcomes that can be modified. Alcohol misuse represents a tremendous burden among these vulnerable youths, and the research to date remains relatively scarce. As such, evidence-based strategies that seek to prevent and delay alcohol use and reduce its harm are very much needed in vulnerable populations, particularly those in the slums [33,34,35,36,37]. Previous research in Western Kenya has demonstrated that substance use interventions for homeless youth would benefit from the integration of community, family, and friends into traditional interventions, such as psychological therapy and community reinforcement approaches [66]. Harm reduction interventions, such as job skills training and supportive housing, may also be effective at decreasing youth alcohol consumption [67]. While traditional approaches used for community-based interventions may need to be adapted in terms of available resources, scope, and the mode of delivery, there are clear and pragmatic evidence-based strategies that can be implemented and evaluated to address the tremendous burden of alcohol and harm in this and similarly vulnerable populations in low resource settings, such as slums.

## 5. Conclusions

In this study we examined factors associated with alcohol-related violence among youth living in the slums of Kampala. The findings show that parental alcohol use, childhood abuse, homelessness, and frequent heavy drinking, drunkenness, and problem drinking measured by the CAGE were significantly associated with alcohol-related violence. Frequent drinking, heavy drinking, and drunkenness also mediated the association between homelessness and alcohol-related violence. Additionally, there were no differences in alcohol-related violence between males and females. Evidence-based strategies are urgently warranted to prevent and delay alcohol use and harm in this population.

## Figures and Tables

**Table 1:** Demographic characteristics and psychosocial correlates of alcohol-related violence among youth living in the slums of Kampala who reported consuming alcohol, 30.5% (*n* = 346).

	Alcohol-related Violence *n* = 158 (45.7%)	No alcohol-related Violence *n* = 188 (54.3%)	Total Sample	Chi-Square, *(df)*, *p*-value
Gender				
Male	79 (50.0%)	76 (40.3%)	155 (44.8%)	3.18, (*1*), *p* = 0.07
Female	79 (50.0%)	112 (59.6%)	191 (55.2%)	
Age, median (IQR) *	17.0 (1.0)	17.0 (2.0)	17.0 (2.0)	*p* = 0.38
Education				2.60, (*2*), *p* = 0.27
<Primary	63 (40.1%)	60 (32.3%)	123 (35.9%)	
Completed primary	34 (21.7%)	41 (22.0%)	75 (21.9%)	
>Secondary	60 (38.2%)	85 (45.7%)	145 (42.3%)	
Parental alcohol use				4.55, (*1*), *p* = 0.03
Yes	117 (74.5%)	120 (63.8%)	237 (68.7%)	
No	40 (25.5%)	68 (36.2%)	108 (31.3%)	
Childhood abuse				6.85, (*1*), *p* = 0.009
Yes	87 (55.1%)	77 (41.0%)	164 (47.4%)	
No	71 (44.9%)	111 (59.0%)	182 (52.6%)	
Ever living on the streets (homelessness)				13.87, (*1*), *p* = 0.0002
Yes	78 (49.4%)	56 (29.8%)	134 (38.7%)	
No	80 (50.6%)	132 (70.2%)	212 (61.3%)	
Problem drinking (CAGE)				16.87, (*1*), *p* < 0.0001
Yes	94 (59.9%)	70 (37.6%)	164 (47.8%)	
No	63 (40.1%)	116 (62.4%)	179 (52.2%)	
Alcohol use frequency				23.08, (*1*), *p* < 0.0001
<4 times a month >5 a month	57 (36.1%) 101 (63.9%)	116 (62.0%) 71 (38.0%)	173 (50.1%) 172 (49.9%)	
Number of drinks per day				27.7, (*1*), *p* < 0.0001
1–2 drinks	65 (41.1%)	129 (69.4%)	194 (56.4%)	
3 or more drinks	93 (58.9%)	57 (30.7%)	150 (43.6%)	
Binge drinking days past				23.73, (*1*), *p* < 0.0001
month				
0 days	26 (16.6%)	76 (40.6%)	102 (29.7%)	
1 or more days	131 (83.4%)	111 (59.4%)	242 (70.4%)	
Number of days drunk				13.97, (*1*), *p* = 0.0002
0 days	17 (10.8%)	50 (26.7%)	67 (19.4%)	
1 or more days	141 (89.2%)	137 (73.3%)	278 (80.6%)	

* Wilcoxon Rank-Sum Test conducted for differences in ages due to the non-normality of the age distribution. CAGE = Cut, Annoyed, Guilty, Eye.

**Table 2:** Alcohol-related violence and problem drinking among youth living in the slums of Kampala, Uganda (*n* = 344).

Because of Your Own Alcohol Use, How Often during the Last 12 Months Have You Experienced the Following:	Non-problem Drinkers by CAGE Scores, *n* = 180 (52.3%)	Problem Drinkers by CAGE Scores, *n* = 164 (47.7%)	Total (*n* = 344)	Chi-Square, *df*, *p*-Value
Got in a fight				
Never	116 (64.8%)	70 (42.7%)	186 (54.2%)	16.87, (*1*), *p* < 0.0001
1 or more times	63 (35.2%)	94 (57.3%)	157 (45.8%)	
Got in an accident				
Never	147 (82.2%)	115 (70.1%)	262 (76.4%)	6.83, (*1*), *p* = 0.009
1 or more times	32 (17.9%)	49 (29.9%)	81 (23.6%)	
Had serious problems with your parents				
Never	140 (78.2%)	90 (54.9%)	230 (67.1%)	21.09, (*1*), *p* < 0.0001
1 or more times	39 (21.8%)	74 (45.1%)	113 (32.9%)	
Had serious problems with your friends				
Never	110 (61.5%)	63 (38.4%)	173 (50.4%)	18.17, (*1*), *p* < 0.0001
1 or more times	69 (38.6%)	101 (61.6%)	170 (49.6%)	
Was a victim of robbery or theft				
Never	146 (81.6%)	111 (67.7%)	257 (74.9%)	8.78, (*1*), *p* = 0.003
1 or more times	33 (18.4%)	53 (32.3%)	86 (25.1%)	
Had trouble with the police				
Never	152 (84.9%)	126 (76.8%)	278 (81.0%)	3.64, (*1*), *p* = 0.06
1 or more times	27 (15.1%)	38 (23.2%)	65 (19.0%)	
Had to go to a hospital				
Never	162 (90.5%)	122 (74.4%)	284 (82.8%)	15.60, (*1*), *p* < 0.0001
1 or more times	17 (9.5%)	42 (25.6%)	59 (17.2%)	

* Missing data (*n* = 2) were due to participants not completing the CAGE questionnaire.
